# From Millimeters to Microns: A Hybrid Strategy for Reconfigurable Liquid‐Crystal Patterning

**DOI:** 10.1002/smtd.202501892

**Published:** 2026-02-18

**Authors:** Adithya Pradeep, Yunuen Montelongo, Jun‐Seok Ma, Zhiyu Xu, Tianxin Wang, Ji Qin, Camron Nourshargh, Martin J. Booth, Steve J. Elston, Stephen M. Morris

**Affiliations:** ^1^ Department of Engineering Science University of Oxford Oxford UK; ^2^ Nature Sciences Research Institute KAIST Yuseong‐gu Daejeon Republic of Korea

**Keywords:** direct laser writing, high‐resolution structures, large‐area fabrication, liquid crystals, two‐photon polymerization

## Abstract

Programmable patterning of nematic liquid crystals (LCs) enables spatially encoded optical functionality, but existing approaches often face trade‐offs between patterned area, feature fidelity, dimensionality, and device integration. Many methods rely on surface alignment, electrode patterning, or global illumination, limiting local addressability, depth control, or scalability. Field‐assisted polymerization of reactive mesogens offers a means to capture designed LC director configurations as permanent, structurally encoded profiles. Here, we report a hybrid strategy combining wide‐field one‐photon polymerization (1PP) for rapid, large‐area templating with two‐photon polymerization direct laser writing (2PP‐DLW) for localized, maskless microstructuring with depth control. This decoupling of patterned area from feature fidelity allows multiple, spatially co‐located director profiles to be encoded within a single glass cell, enabling voltage‐selective visibility and reconfigurable optical responses. Local 2PP‐DLW features define confinement boundaries and deterministic defect nucleation and guidance, while 1PP establishes the global architecture with high throughput. Because patterning is encoded in the polymer rather than electrode geometry, complex profiles can be realized using uniform electrodes. In a nematic Pi‐cell, this approach enables controlled defect channeling, programmable topological transitions, and multistate optical patterns, offering a scalable route to high‐fidelity reconfigurable LC micro‐optics.

## Graphical abstract

A hybrid approach combining wide‐field exposure and direct laser writing is used to sculpt responsive polymer architectures in nematic liquid crystals. This method enables voltage‐tunable textures, channeling of topological defects, and hierarchical resolution control. The patterned structures remain stable across applied fields, offering a reconfigurable platform for next‐generation photonic and display devices.

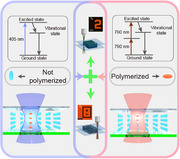

## 1 Introduction

Liquid crystals (LCs) are a unique class of anisotropic molecules, characterized by their birefringence and the ability to reorient under an applied voltage, making them valuable for various electro‐optic applications [1, 2, 3, 4]. LC phases exhibit a distinct combination of order and mobility: while their constituent molecules are sufficiently disordered to impart softness, they maintain varying degrees of order, creating a versatile state of matter  [5, 6]. The addition of a reactive mesogen to a nematic LC results in distinctive advantageous properties such as creating composite materials [7] and forming defined pretilt angles in multiple directions as is the case for a patterned vertical aligned (PVA) LC device [8]. Furthermore, polymer‐stabilization can lead to faster switching speeds, higher transmission and better image quality compared to vertical aligned displays without polymer stabilization [9] as well as improving the optical quality of smart windows [10, 11]. Notably, reactive mesogens also enable advanced patterning processes, which involve manipulating the molecular arrangement of LCs to achieve specific optical and morphological characteristics  [12, 13, 14].

Various LC patterning techniques reported to date include photoalignment [15, 16, 17], dynamic polarization patterning [18], electro‐optical methods [19], mechanical patterning [20, 21], and thermal patterning [22]. Beyond rigid cells, related fabrication routes for LC elastomer architectures, including fiber and cylindrical actuator formats produced using soft tubular molds or centrifugal processing, illustrate practical pathways toward flexible, encapsulated LC‐based form factors [23, 24]. These techniques are integral to advancing applications such as display technologies [25] and sensors [26]. The significance of LC patterning lies in its ability to enhance device performance [27], offering improvements in optical properties such as brightness [28] and responsiveness in electronic displays, while also enabling novel functionalities in biosensing [29, 30].

Polymerization‐based approaches overcome key limitations of purely field‐driven LC patterning by converting a transient, voltage‐induced director configuration into a mechanically stabilized template, which suppresses spatial non‐uniformities associated with fringing fields and improves repeatability [31]. Importantly, polymer stabilization can also enable genuinely bistable operation, in which distinct optical states remain stable at 0 V, reducing or eliminating the need for a holding voltage [32]. Finally, two photon polymerization enables true 3D confinement and localized placement of internal features within the LC volume, providing a route to 3D alignment control that is difficult to achieve with planar electrode addressing alone [33]. Moreover, polymerization‐based structuring provides improved local addressability because the patterned regions are defined by where polymer is written rather than by electrode pitch, allowing features to be introduced only where required without fringing‐field cross‐talk and enabling complex textures to be encoded using uniform electrodes rather than patterned electrode architectures.

In patterned polymer‐walled geometries, electrically driven topological domain growth can produce a pseudo‐hysteretic response that enables bistability and allows a selected topological state to be locked‐in and retained within the defined region [34]. In established polymerization‐based patterning routes such as POLICRYPS, the polymer morphology is typically defined by interference or holographic exposure, which naturally favors periodic grating‐type structures and can restrict the range of arbitrary, freeform patterns that can be encoded without additional patterning steps [35]. As a result, deterministic placement of isolated features, non‐periodic confinement boundaries, or locally varying pattern geometries within an otherwise uniform device is difficult to achieve using POLICRYPS alone. The resulting polymer network or microstructure nonetheless effectively pins domain boundaries and defect pathways, increasing the stability and long‐term permanence of the patterned texture while the surrounding, non‐polymerized LC can remain electrically switchable. In practice, this enables non‐volatile or reduced‐hold‐voltage operation and more reproducible reconfiguration cycles compared with purely field‐defined patterns.

Other patterning processes such as electrochromic patterning, photoalignment, and electric‐field‐driven LC patterning each offer complementary strengths for spatial structuring, but they differ markedly in achievable spatial resolution, pattern scale, reconfigurability, dimensionality, stability, and fabrication throughput (summarized in Table 1). Electrochromic approaches support large‐area patterning from millimeter to tens‐of‐centimeter scales with bistable operation at 0 V, but lateral feature sizes are more typically limited to the ∼10–100 µm range, and performance can be constrained by ionic transport, electrode definition, and cycling or ageing effects [36, 37]. Photoalignment enables high‐quality surface patterning with lateral resolutions of ∼0.5–5 µm over areas ranging from ∼100 µm to ∼1 cm, but the control remains confined to 2D substrate boundary conditions and is generally static after writing unless re‐exposed [38, 39]. Electric‐field‐driven LC patterning offers fully dynamic, video‐rate reconfiguration, typically in the ∼10–240 Hz range, and can be scaled to panel dimensions, although effective feature fidelity is constrained by electrode pitch and fringing‐field cross‐talk, commonly in the ∼10–20 µm range, and patterns are volatile once the applied electric field is removed [40, 41].

**TABLE 1:** A comparative summary of typical spatial resolution, pattern scale, reconfigurability, out‐of‐plane control, stability and fabrication throughput for hybrid 1PP/2PP structuring (this work), electrochromic patterning [36, 37, 42], photoalignment [38, 39, 43], and purely electric‐field‐driven LC patterning [40, 41, 44].

Method	Hybrid 1PP/2PP	Electrochromic patterning [36, 37, 42]	Photoalignment [38, 39, 43]	Electrical Field‐Driven LCs [40, 41, 44]
**Spatial Resolution**	1.8 to 2.7 µm	10 to 100 µm	0.5 to 5 µm (only surface)	10 to 20 µm
**Pattern Scale**	∼10 µm to ∼5 cm	∼50 µm to 10s of cm	∼100 µm to ∼1.1 cm	∼mm to 10s cm
**Reconfigurability**	Predefined multi‐state (≥ 2–3 states; 0–10 V demonstrated)	Reversible (typically 2 states; colored/bleached)	Static after writing (rewriting requires re‐exposure)	Fully dynamic (video‐rate refresh, ∼10–240 Hz)
**3D/Out‐of‐Plane Control**	Yes	No	No (Surface alignment only)	No
**Stability**	Permanent, field ‐stable at any voltage (0–10 V demonstrated here)	Bistable at 0 V (limited by cycling/ageing)	Permanent surface alignment; stability depends on alignment layer and UV or thermal history	Not stable once field is removed
**Fabrication Throughput**	1PP: mm^2^ ‐ min^−1^; 2PP: µm^2^‐mm^2^ h^−1^ (local)	mm^2^—cm^2^ min^−^^1^ (coating/printing)	µm^2^ ‐ mm^2^ min^−1^ (parallel exposure)	panel‐scale (mm^2^ ‐ cm^2^ per process cycle)

In contrast, the hybrid one photon polymerization‐two photon polymerization (1PP–2PP) fabrication strategy used in this paper, combines high‐throughput, parallel one‐photon polymerization for large‐area templating with localized two‐photon polymerization micro‐structuring for precision control, providing volumetric 3D constraints and stable, structurally encoded LC director profiles with lateral feature sizes in the ∼1.8–2.7 µm range. This approach supports predefined multistate operation under applied voltages, with field‐stable behavior demonstrated over the 0–10 V range. By decoupling patterned area from local feature fidelity, the hybrid workflow enables hierarchical patterns spanning ∼10 µm to centimeter scales within a single device, which is difficult to achieve using purely surface‐defined or electrode‐defined patterning approaches.

Related strategies have employed holographically generated or tiled light fields to enable parallel laser writing using spatial light modulators, allowing simultaneous patterning at multiple lateral positions or depths [45]. While such holographic parallelization can substantially increase throughput, extending these methods to large‐area or highly complex, user‐defined patterns typically requires sophisticated optical architectures, high‐performance spatial light modulators, and iterative feedback or optimization procedures to maintain uniformity and stability across all focal sites. As a result, experimental complexity and system cost increase significantly as pattern size, dimensionality, or flexibility are scaled.

Therefore, in view of the limitations associated with electrochromic, photoalignment and purely electric‐field‐driven approaches, polymerization‐based methods have emerged as a promising route for spatial patterning of LCs, as they can convert field‐induced configurations into structurally stabilized templates. In particular, two‐photon polymerization direct laser writing (2PP‐DLW) enables high‐fidelity, maskless patterning of microstructures with depth control and has been widely adopted for creating high‐resolution optical features [46, 47, 48]. Partial locking can also be achieved when polymerization is performed in a defined geometry that pins LC domain boundaries and stabilizes a selected region of the texture [34]. However, because 2PP‐DLW is a serial, voxel‐by‐voxel process, its main limitation is the comparatively low fabrication throughput, which motivates hybrid strategies that reserve 2PP for local high‐resolution regions while using parallel methods for large‐area templating.

The patterning of nematic LCs with a polymer network is achieved through free radical polymerization [49], initiated by a photoinitiator that generates free radicals upon exposure to light. During this process, phase separation occurs, in which the growing polymer chains demix from the LC matrix and spatially segregate, as confirmed by infrared (IR) spectroscopy, scanning electron microscopy (SEM), and neutron scattering [50]. These free radicals drive the polymerization process by propagating through chain‐to‐chain interactions and progressing to the termination step, thereby locking the director structure into the desired orientation for the patterning process. The composite birefringence of the polymer–LC region closely matches that of the LC, with the polymer's extra contribution remaining small, with only weak temperature dependence [51].

One‐photon polymerization (1PP) is enabled by linear absorption [52], allowing large areas to be exposed in parallel so that millimeter to centimeter scale templates can be defined in a single step. By contrast, two‐photon polymerization direct laser writing (2PP‐DLW) relies on nonlinear absorption [53] confined to the focal volume, enabling maskless, localized micro‐structuring with depth control and high feature fidelity, albeit with reduced throughput due to serial writing. This complementarity has motivated hybrid process flows in which a rapid, lower complexity exposure defines the macroscopic architecture while 2PP is reserved for features that require precise local control. For example, hybrid workflows combining 2PP with conventional ultraviolet (UV) lithography have been used in other application areas to couple high‐resolution microfabrication with rapid formation of larger support structures and improved handling [54].

Here in this paper an experimental method is presented that can be used for manipulating the orientation of LCs across a range of length scales to create various patterns and features suitable for electro‐optic applications. The approach employs an optical system designed to polymerize and lock the director orientation into specific patterns through one‐photon polymerization (1PP) before 2PP is then utilized to produce finer, higher‐resolution polymer structures. By combining 1PP, enhanced to achieve comparable resolution through spherical aberration correction, with 2PP‐DLW, this method enables the fabrication of larger‐scale, high‐resolution patterns. The resulting patterned structures have significant potential for various advanced electro‐optic applications.

Additionally, this work also demonstrates how this hybrid patterning approach can be applied to Pi‐cell geometries to create voltage‐responsive patterns building upon our previous work on leveraging the electric field induced topological transitions inherent to Pi cells [34] with patterned structures that can guide the movement of defect lines and enable the formation of distinct, reconfigurable textures. While the underlying mechanism has been established [55], the integration of both bulk (one photon) and localized (two photon) polymerization enables simultaneous control over larger area patterning and finely detailed features, allowing the structured guidance of topological domains into application relevant configurations.

## 2 Results

### 2.1 One and Two‐Photon Polymerization

To enable scalable yet high‐resolution patterning in LCs, a hybrid fabrication strategy is employed by combining 1PP with 2PP‐DLW. Each technique offers distinct advantages: 1PP allows rapid exposure over millimeter‐scale areas due to wide‐field illumination, while 2PP‐DLW provides submicron resolution through localized voxel‐by‐voxel writing. The integration of these methods enables the fabrication of LC devices that incorporate both large‐area alignment and intricate structural detail.

**FIGURE 1 (a):**
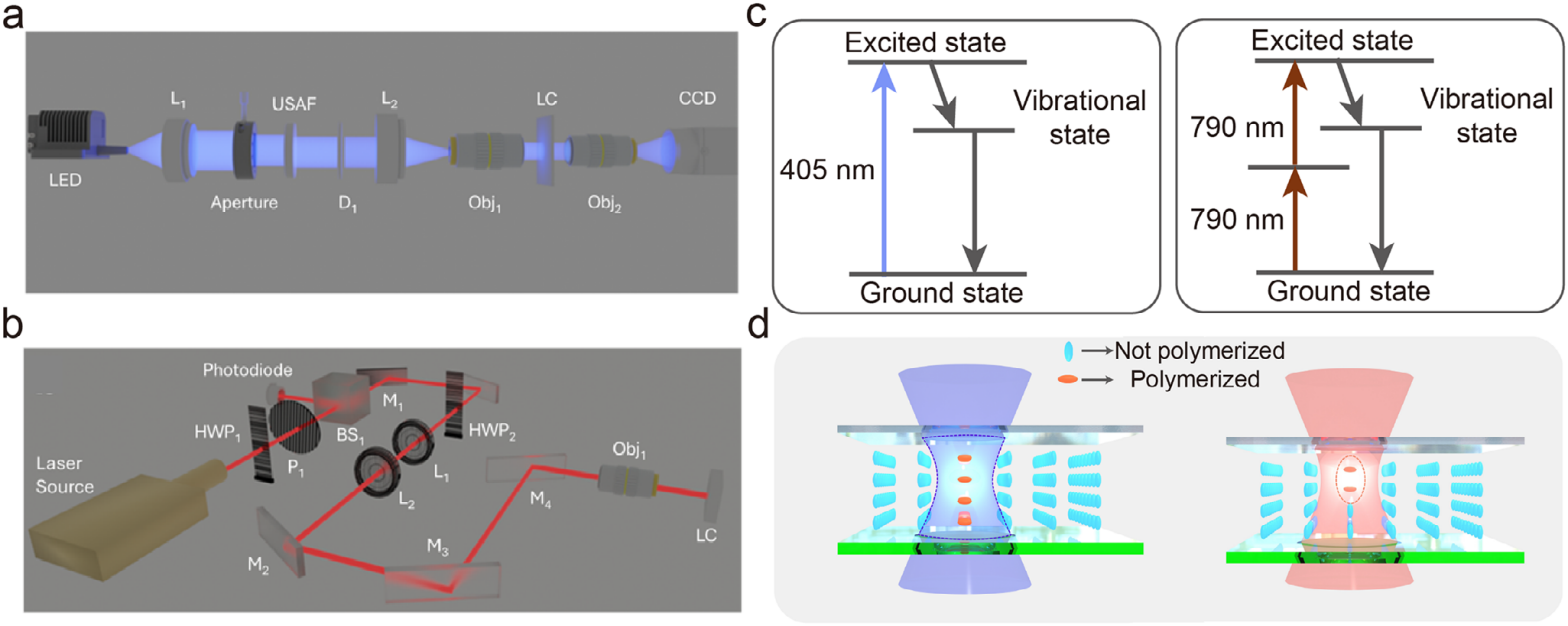
Schematic of the bulk projection system used for one‐photon polymerization. The components from left to right are as follows: a fiber‐coupled LED emitting at 405 nm, a plano‐convex lens (*f* = 25.4 mm), an aperture, a USAF test target, a diffuser, another plano‐convex lens (*f *= 50 mm), a 2.5×/0.07 microscope objective, and the liquid crystal (LC) cell for patterning. The imaging system, placed beyond the LC cell, ensures a well‐focused image of the pattern. (b) Diagram of the laser writing system. The laser (Mai Tai Ti:Sapphire) passes through a half‐wave plate, a polarizer, and a beam splitter. Part of the beam is diverted to a photodiode for monitoring the incident power, while the remainder is directed through another half‐wave plate. The beam then passes through two lenses (*f* = 80 mm and *f* = 300 mm), before being redirected by mirrors to a 20×/0.45 microscope objective. (c) Energy‐level diagrams for one‐photon and two‐photon polymerization. (d) The schematics illustrate the difference between creating voxels for localized patterning (two‐photon) and patterning the entire LC plane (one‐photon), including the LC layer between the top and bottom glass substrates.

In the 1PP configuration (Figure 1a), a 405 nm LED light source, coupled through a microfiber cable, was directed onto a plano‐convex lens for collimation. On its own, the plano‐convex lens produces spherical aberrations because peripheral rays are focused closer to the lens than paraxial rays, resulting in a blurred or distorted focus. To mitigate this effect, the collimated light was first reduced in diameter using an aperture to limit peripheral rays, and then relayed through a 2.5×/0.07 NA microscope objective. This two‐lens arrangement (plano‐convex lens combined with a low‐NA objective) suppresses spherical aberrations compared to the plano‐convex lens alone, restoring edge contrast and improving pattern sharpness, as confirmed in Supplementary Figure S1b. After optimization, the light passed through a 1951 USAF resolution test target, projecting the desired pattern onto the LC device. The projected light field was then captured using an imaging system composed of an objective lens and a CCD camera, both focused on the LC layer to ensure precise alignment. To enhance resolution, the LC cell was mounted on a translation stage that scanned along the x, y, and z axes, enabling full‐area exposure of the projected pattern.

For high‐resolution patterning, a custom 2PP‐DLW system was employed (Figure 1b). Ultrafast femtosecond laser pulses (100 fs, 80 MHz) at a wavelength of 780 nm were generated from a Ti:Sapphire laser. The beam passed through a half‐wave plate and a polarizer, followed by a beam splitter to control intensity. It was then routed through a second half‐wave plate and a series of lenses before being focused into the LC cell using a 20×/0.45 NA microscope objective. Although higher NA objectives, such as 1.4 oil immersion, provide greater resolution, they significantly increase fabrication time and reduce throughput. An NA of 0.45 was therefore selected to achieve a balance between resolution and processing efficiency.

The fabrication time, *t* for 2PP‐DLW can be estimated using the expression [56]:

(1) $$t=\frac{xyzF}{Rv}$$

where *x,y,z* are the polymer structures’ width, length, and height, *R* is the voxel volume, *v* is the scanning speed, and *F* the fill factor (the ratio of polymerized volume to non‐polymerized volume in the structure design). This expression yields the fabrication time normalized per unit length. For a structure measuring 500 µm × 500 µm × 100 µm, with a fill factor of 50%, a voxel volume of 0.14 µm^3^ (with numerical aperture (NA) = 1.4), and a scanning speed of 100 µm/s, the fabrication time is estimated to be ∼ 9 × 10^5^ s (over 248 h).

A key advantage of using these two methods (1PP and 2PP) in LCs is the ability to match the refractive index of the patterned polymer structures with that of the non‐polymerized LC [57]. This property enables the creation of LC patterns that are optically visible at one applied voltage and become cloaked at another [58]. Additionally, for the 2PP DLW, due to the high pulse energy and short pulse intervals, two‐photon absorption within the LC can polymerize the region defined by the voxel, allowing the fabrication of polymer structures near the diffraction limit [59, 60].

The differences in excitation mechanisms between the two techniques are illustrated in Figure 1c. In 1PP, polymerization occurs through linear absorption of a single photon, resulting in uniform polymerization throughout the entire illuminated region. By contrast, 2PP involves nonlinear absorption of two photons of near‐infrared light in rapid succession. Since the absorption rate is proportional to the square of the photon flux, polymerization is confined to a small volume at the beam's focal point. This spatial confinement enables high‐resolution structuring with minimal off‐target effects.

As shown in Figure 1d, the polymerized regions differ between the two methods: in 1PP, the beam path results in continuous polymerization across the illuminated plane, while in 2PP, discrete voxel‐sized features are created at the focus. This distinction allows 2PP to structure polymer networks within specific subregions of the LC cell, whereas 1PP is used for rapid definition of broader domains. The combined approach supports hierarchical patterning, with 2PP introducing intricate features and 1PP providing macroscopic pattern structure.

The distinction is pivotal for patterning, as 2PP enables precise, high‐resolution structuring of the polymer network in the nematic LC within selected regions. Simultaneously, the large‐scale features can be efficiently created through bulk polymerization using the 1PP process. The combination of both techniques provides a powerful approach to generating intricate, high‐resolution structures alongside large‐scale patterns, advancing the capabilities of LC device fabrication. Supplementary Figure S2 and Supplementary Figure S3 showcase distinct patterns generated using the bulk projection (1PP) and laser writing (2PP) configurations, respectively, demonstrating the versatility and complementary nature of these optical techniques for fabricating diverse LC patterns.

**FIGURE 2:**
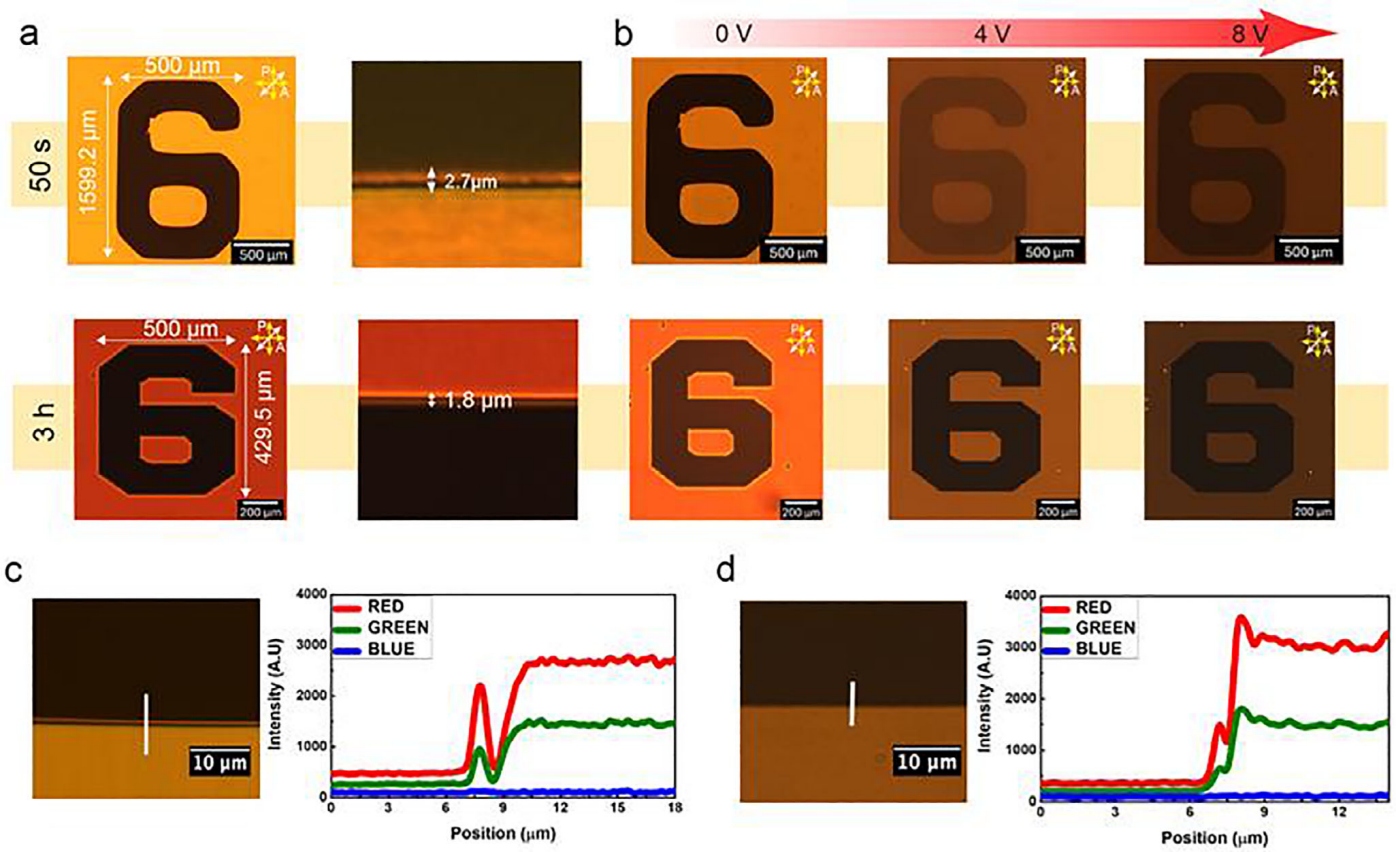
Comparison between one‐photon and two‐photon polymerization patterning. (a) Larger patterns (dimensions: 1135 µm × 1599 µm) were formed using bulk (1PP) projection with an exposure time of 50 s. Smaller patterns (dimensions: 365 µm × 430 µm) were formed using laser writing (2PP) with a 3‐h fabrication time. The cross‐sectional line width highlights the intensity fringes at the pattern edges, showing resolutions of ≈3 µm for 1PP and ≈2 µm for 2PP. (b) The alphanumeric “6” imprinted into an LC sample (regions where the LC is locked into a homeotropic alignment) formed using 1PP (top) and 2PP (bottom) at varying applied voltages (0, 5, and 10 V) post‐fabrication. (c) Line scan of the 1PP fabricated pattern, displaying broad fringe spacing and gradual intensity variation, consistent with lower patterning resolution (d) Line scan of the 2PP fabricated pattern, showing sharp intensity transitions and tightly confined fringes corresponding to high spatial resolution.

To demonstrate the capabilities of both patterning techniques, a comparison between 1PP and 2PP structuring was conducted. Figure 2a compares patterns produced by 1PP using the bulk projection system and 2PP using direct laser writing. In the one‐photon approach, the 405 nm LED illuminated a nematic LC cell (composed of E7 [79 wt.%], RM257 [20 wt.%], and IR819 [1 wt.%]) for 50 s, projecting the “6” alphanumeric (from a USAF resolution test target) onto the cell. This process formed a large‐scale feature approximately 1600 µm (height) × 1135 µm (width). The polymer locking process was achieved by first subjecting the LC to 10 V to align the director homeotropically, after which a region of LC resembling the pattern from the USAF was polymerized.

Bright and dark interference fringes were observed at the boundaries of the polymer‐stabilized regions, exhibiting a spacing of ≈3 µm. These fringes arise from diffraction effects at sharp optical transitions and indicate the resolution limit of the 1PP system. After polymerization at 10 V, representative polarizing optical microscopy (POM) images in Figure 2b at 0, 4, and 8 V demonstrate that the pattern remains unchanged upon the application of voltage post‐fabrication, indicating that the director configuration has been successfully locked in place by polymer stabilization and does not undergo further switching.

In contrast, the 2PP‐DLW method required approximately 3 h to fabricate the same pattern at reduced dimensions of approximately 430 µm (height) × 365 µm (width). Despite the slower throughput, the laser writing approach produced finer polymer features, as evidenced by inter‐fringe spacing of ≈2 µm at the pattern edges. POM images taken at voltages from 0 V to 8 V after fabrication likewise confirmed that the laser‐written structures remain stable across a range of applied voltages, indicating effective polymer stabilization in the irradiated regions. Beyond structural stability, the patterned regions in both the 1PP‐ and 2PP‐fabricated areas also exhibit dynamic modulation of visibility under applied voltage, as demonstrated by the optical cloaking effect shown in Supplementary Figure S4 and as reported previously [58]. While the 1PP method rapidly produced millimeter‐scale patterns within 50 s, 2PP required several hours to generate significantly smaller features. This highlights a clear trade‐off between fabrication speed and spatial resolution.

Figure 2c,d provides a quantitative comparison of the resolution achieved by each technique using intensity line scans across the patterned edges. These scans capture the diffraction fringes generated at refractive‐index or director‐alignment discontinuities. While both projection (1PP) and laser writing (2PP) methods produce such fringes, the spacing and sharpness of the fringes serve as a direct measure of resolution. The bulk‐projected pattern exhibits broader fringes with slowly varying intensity, consistent with a resolution limit of ≈3 µm. By contrast, the laser‐written pattern shows closer fringe spacing of ≈2 µm and steeper edge transitions, indicating a sharper interface.

In addition to fringe spacing, the transition width, defined as the full width at half maximum (FWHM) of the intensity rise, is narrower in the laser‐written case. This reflects tighter spatial confinement of the polymerized network and greater fidelity of the written structure. These results validate the superior spatial resolution of 2PP compared to 1PP. The red and green intensity traces in Figure 2c,d highlights these clear transitions, whereas the blue trace remains nearly constant, consistent with the use of a bandpass filter that blocks wavelengths below 552 nm. This prevents unintentional polymerization by blue light. Notably, the laser‐written “6” demonstrates a narrower optical profile and sharper edges than the 1PP‐generated counterpart, consistent with the voxel‐level control offered by 2PP‐DLW.

The above outline the key performance metrics that uniquely favor 1PP and 2PP. A central distinction is spatial resolution, as highlighted in Figure 2a. In our system, 2PP enables the fabrication of highly resolved microstructures with a measured spatial resolution of 1.8 µm, which is essential for reliably producing complex features with critical dimensions below about 2 µm. In contrast, 1PP is uniquely favored by large‐area throughput, defined as the patterned area per unit time. Because 1PP is a parallel exposure technique, it enables rapid patterning over millimeter to centimeter‐scale areas with a low cost per unit area, making it well suited for defining the global device architecture. Beyond lateral resolution, 2PP provides two additional capabilities that are difficult to achieve with 1PP: local, maskless addressability and out‐of‐plane structuring. Here, out‐of‐plane structuring refers to the ability to place polymer features with controlled height and depth within the cell gap (LC layer), enabling 3D constraints such as pillars, walls and embedded pinning elements that shape the LC director field throughout the volume rather than only at the substrates. Consequently, the hybrid 1PP–2PP workflow decouples scale from resolution by using 1PP for fast, large‐area templating and reserving 2PP for regions that require high‐fidelity micro‐structuring and localized 3D control.

### 2.2 Combining 1PP and 2PP Patterning

**FIGURE 3:**
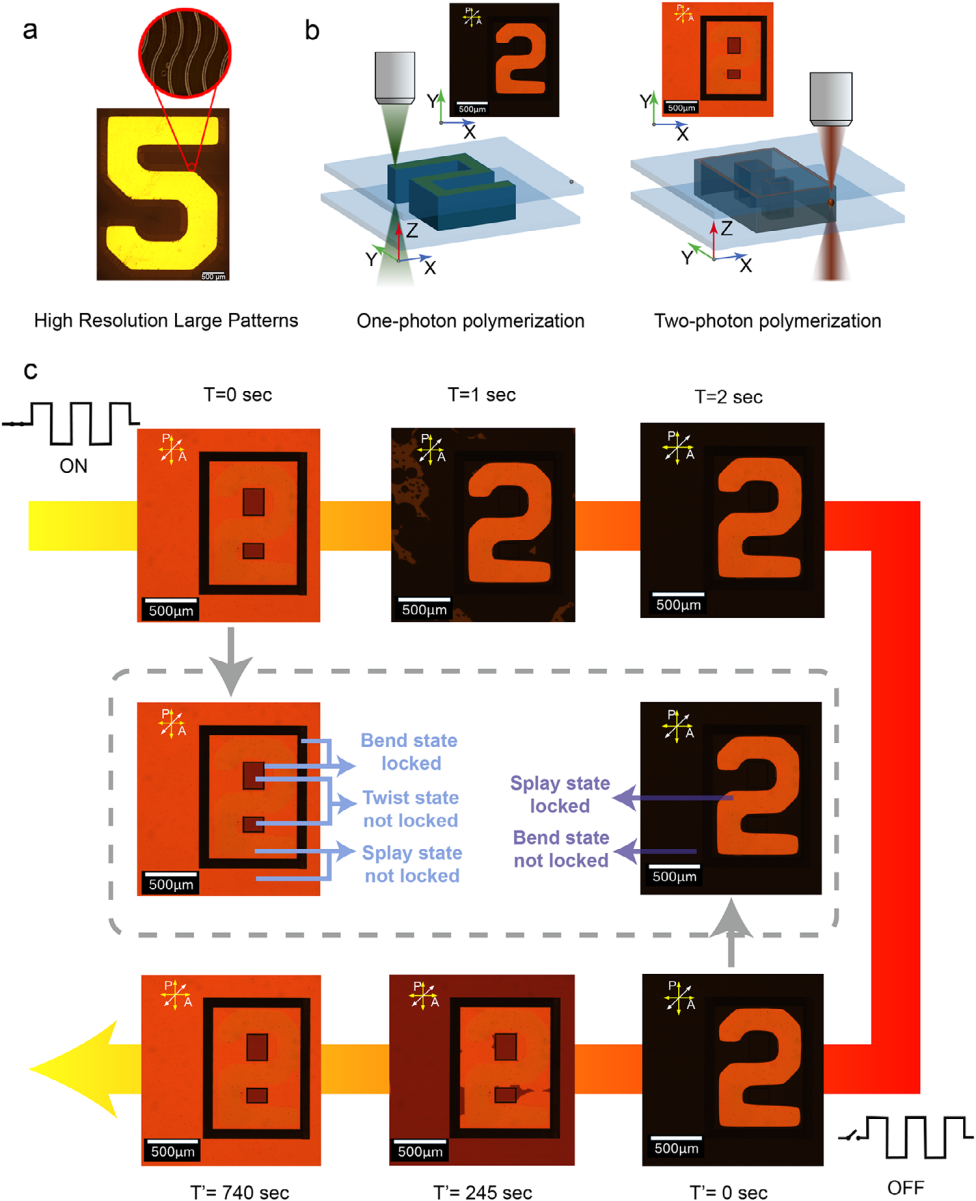
Formation of various patterns using a combination of laser writing and bulk projection. (a) High‐resolution pattern ("5") created in a planar‐aligned polymerizable nematic LC cell with anti‐parallel rubbing, utilizing the bulk projection system (1PP) with detailed hairline resolution written with laser writing (2PP). (b) Demonstration of creating distinct topological structures ("2" and “8”) in the same LC cell: 1PP at 0 V (left) and 2PP at 10 V (right) show voltage‐dependent patterning in a parallel‐rubbed polymerizable nematic LC (pi)‐cell. (c) Transformation of the written patterns under applied voltage, where the combined use of bulk projection (1PP) and laser writing (2PP) directs the evolution of defect states. Time stamps annotate the transitions for both switching directions (0 to 10 V and 10 to 0 V). The scale bar in all images represents 500 µm.

A hybrid patterning strategy combining 1PP and 2PP was then employed to produce both large‐scale and high‐resolution features within the same LC device. This approach leverages the strengths of each method: namely, rapid wide area structuring via 1PP and then high resolution and precise patterning via 2PP. Figure 3 illustrates example demonstrations of this combined approach. In Figure 3a, a high‐resolution “5” pattern was created in a planar‐aligned nematic LC cell with antiparallel rubbing, using 1PP to define the macroscopic shape. Fine polymer features measuring approximately 100 µm in length, representing sub‐patterns within the main shape, were then subsequently patterned using 2PP. This demonstrates that the hybrid method enables the formation of larger‐area patterns while simultaneously embedding fine structural details that enhance patterning functionality. An example POM image of the device, captured under a 10 V read voltage, confirms that the patterned structure remains optically visible and stable within the LC cell.

In a second demonstration, shown in Figure 3b,c, the hybrid patterning technique was applied to a nematic Pi‐cell (parallel rubbed alignment layers) capable of supporting multiple voltage‐dependent topological regimes [34]. The schematic in Figure 3b illustrates the spatial layout of regions of the LC patterned (polymerized network) by 1PP and 2PP, with the large‐scale “2” pattern defined by 1PP and the guiding channels and confinement boundaries created using 2PP. A “2” shape was first fabricated using 1PP when no voltage was applied to the LC. Polymer walls were subsequently written using 2PP at 10 V to lock the bend state, thereby creating guiding corridors for defect motion. It is known that polymer walls written in the bend state at high voltage can lock a metastable twist configuration [34], and that defect lines generated during the twist‐to‐splay transition can be efficiently channeled when the corridors are bounded by bend‐locked walls [55]. An example of guided defect motion within bend‐locked channels is provided in Supplementary Figure S5, where 1PP‐written polymer walls define the channel boundaries and a localized 2PP‐written feature acts as an internal guide that directs the nucleation and propagation of the splay defect wall under voltage modulation. Following these established principles, defect‐line channels and confinement walls were written at 10 V. At 10 V, two interior rectangles (270 µm × 210 µm and 155 µm × 210 µm) and an outer frame (1070 µm × 1405 µm) were also polymerized via 2PP to create tunable confinement zones.

A defect‐nucleation line was introduced at 0 V, and upon switching the voltage from 10 to 0 V, the resulting defect propagated through the unpolymerized region while being guided by the 2PP‐defined boundaries. Figure 3c labels the regions that are permanently locked by polymerization and those that remain switchable according to their director orientations and states. This allowed controlled reorganization of the LC director and the associated optical phase profile, where the visible pattern transitioned from a ‘2’ to an ‘8’ under crossed polarizers as the applied voltage was tuned from 10 to 0 V, due to director reorientation in the unlocked, nonpolymerized zones. Figure 3c also shows the voltage‐dependent evolution of the pattern, with time‐stamped frames for the 0 to 10 V and 10 to 0 V steps showing the transition of the optical appearance between ‘2’ and ‘8’. The demonstration highlights the utility of hybrid patterning by channeling defect lines and enabling voltage‐responsive reconfiguration, with polymer‐stabilized regions maintaining alignment and unpolymerized regions undergoing dynamic transitions. In the present work, the structural resolution is found to be limited primarily by optical readout and imaging rather than by polymerization kinetics. Accordingly, 1PP is used for rapid, large‐area templating, while 2PP is used for localized, high‐fidelity micro‐structuring where precise placement is required. In practice, alignment can be affected by substrate tilt and local height variations, which shift the 2PP focal plane relative to the 1PP‐written template and can translate into micron scale placement errors over millimeter‐scale distances. This is mitigated by performing a raster scan to measure the tilt and height map prior to writing and by applying focus correction during 2PP, which improves registration and reduces stitching artefacts at the 1PP–2PP interface.

This hybrid scheme enables both spatial and temporal control over LC patterning. 1PP is used to rapidly define large‐scale geometries, while 2PP introduces fine structural details and spatially localized voltage responsiveness. By combining these approaches, the method allows precise guidance of defect motion and facilitates voltage‐triggered topological transformations. This dual‐mode capability highlights the system's potential for reconfigurable and multistate optical elements. Supplementary Videos 1 and 2 illustrate the behavior, showing how the channelized structure governs defect propagation and how splay‐to‐bend transitions evolve under varying voltages when viewed between crossed polarizers.

## 3 Conclusions

This work presents a versatile hybrid strategy for fabricating polymer network patterns within polymerizable LCs by integrating 1PP with 2PP‐DLW. By leveraging the complementary advantages of these two techniques, it becomes possible to simultaneously achieve rapid fabrication over larger areas as well as high‐resolution structuring of the polymer network within the same LC device. The 1PP method enables rapid projection of millimeter‐scale features using bulk illumination, making it ideal for generating device‐scale patterns efficiently. In contrast, 2PP offers sub‐micron spatial precision and voxel‐level control, which is essential for defining intricate polymer features such as hairline boundaries, defect pathways, or voltage‐tunable elements.

The proposed hybrid 1PP–2PP approach is naturally suited to device classes that already employ rigid sandwich‐cell architectures and benefit from spatially patterned phase and birefringence. Examples include electrically tunable diffractive elements (polarization gratings, Dammann gratings and switchable beam splitters), reconfigurable phase plates and wavefront‐shaping components for adaptive optics, and tunable micro‐optics such as microlens arrays, Fresnel zone plates and other lensing elements, as well as holographic and beam‐steering structures, where polymer‐defined confinement improves feature fidelity and repeatability. It can also generate arbitrary greyscale optical patterns, where the local intensity or phase retardation is set by spatially programmed birefringence, analogous to the multi‐level topological state encoding demonstrated in a nematic Pi‐cell boundary locking [34]. The ability to combine large‐area templating with localized high‐resolution structuring also enables optical security and authentication elements, in which high‐density, multiscale birefringent patterns and defect‐guided features provide robust, difficult‐to‐replicate signatures. In addition, polymer‐walled confinement together with localized 2PP‐written nucleation provides a route to topological memory concepts, in which selectable defect or domain states can be written, stabilized and read out as multistate pixels within a reconfigurable LC cell.

Although the demonstrations presented in this paper use a rigid glass sandwich cell to maximize optical quality and thickness control, the polymer template could be fabricated first and integrated into alternative form factors, including open‐cell “print first, fill later” configurations and, in principle, flexible stacks via printing on thin substrates or lamination [61]. Beyond the “print first, fill later” assembly, the same strategy can be implemented by incorporating the patterned polymer layer directly into the cell build; for example, by fabricating the polymer features on one substrate and then closing the cell and filling under vacuum or capillary action using standard LC assembly processes. In open‐cell geometries, the patterned surface can be contacted to an LC reservoir and subsequently encapsulated, while flexible variants could use thin transparent electrode films and barrier layers to maintain thickness control and sealing.

The dual‐mode approach presented here addresses a key limitation in current LC patterning methodologies by bridging the trade‐off between fabrication throughput and structural detail. Importantly, it facilitates the encoding of both global and localized functionalities within a single device. For instance, large‐scale textures can serve as macroscopic optical masks or alignment templates, while microscale 2PP‐written polymer regions can act as confinement boundaries, defect pinning sites, or reconfigurable domains responsive to applied electric fields. It also demonstrates the local addressability and stability of the LC topological regions defined by the polymer walls (Supplementary Figure S5). In particular, the 1PP‐written polymer walls act as guiding boundaries for topological domain growth, while a localized 2PP‐written microfeature serves as the microscopic trigger that seeds nucleation, thereby evidencing the spatially selective control enabled by the hybrid approach.

Multi‐level, out‐of‐plane anchoring geometries are demonstrated by the variable‐height polymer pillar array in Supplementary Figure S6, where depth control and 3D microstructuring are achieved by 2PP. Patterns with topological or defect‐engineered structures are demonstrated in Supplementary Figure S5 and Supplementary Figure S7b. In Figure S5, defect lines are initiated and guided using a localized 2PP‐written feature within a channel defined by polymer confinement, demonstrating hybrid control of nucleation and transport. By contrast, Figure S7b shows defect‐line formation and guidance in a nematic Pi‐cell geometry arising from 1PP‐written polymer walls that lock the surrounding director configuration, enabling reproducible defect localization within the encoded geometry. Building on this, large‐area gradients in polymer network density and effective anchoring strength could in principle be introduced using greyscale 1PP mask exposure, while localized 2PP‐DLW can superimpose fine features for micron‐scale modulation and deterministic defect control. More complex 3D architectures, including aberration‐correcting elements and vortex‐generating phase structures, are also enabled in principle by TPP‐DLW, supporting extension beyond the geometries demonstrated here.

The experimental demonstrations further show that polymer structures fabricated through this hybrid method remain stable under applied voltages, with defect motion and director reorientation confined by the polymerized regions. This stability and controllability are critical for applications in reconfigurable optics, tunable filters, and LC‐based photonic elements [62, 63]. Moreover, the approach spans length scales. 1PP defines large‐area features and global boundaries at millimeter scales with fast processing, while 2PP writes micron‐scale channels and nodes that precisely steer defects. This hierarchical control enables programmed, voltage‐dependent transitions such as splay‐to‐bend switching and guided transport, opening pathways to adaptive LC devices with dynamic functionality. Supplementary Figure S7a shows that 2PP features can be written directly adjacent to a pre‐polymerized 1PP boundary (dashed line) without visible deformation or displacement of the existing polymer wall, indicating that subsequent 2PP exposure does not mechanically disturb previously formed structures. The sharp, unchanged 1PP edge after 2PP writing confirms that director‐field shaping can be added locally by 2PP while preserving the integrity of the pre‐defined 1PP template.

In conclusion, this hybrid patterning scheme not only expands the design space for LC devices but also enables new modes of electro‐optic control. It paves the way for scalable fabrication of advanced LC systems that combine optical complexity, spatial precision, and dynamic responsiveness, with potential applications in displays, sensors, spatial light modulators, and programmable photonic circuits.

## 4 Methods

### 4.1 Liquid Crystal Preparation

A mixture of 79 wt.% E7 (Synthon Chemicals Ltd), 20 wt.% RM257 (Merck), and 1 wt.% IR819 was employed for this research. The resultant blend was introduced into glass cells, which featured a 5 µm gap defined by spacer beads. Filling was performed at a temperature above the mixture's clearing point. Each glass substrate included an indium tin oxide (ITO) layer to permit the application of an external electric field, as well as a rubbed polyimide coating. Electrical connections were made via wires soldered to the substrates. Subsequently, the LC device was mounted into either the 1PP or 2PP optical systems, allowing targeted polymer network formation under controlled voltage.

### 4.2 Polarized Optical Microscopy

An Olympus BX51 polarizing optical microscope (POM) equipped with a QImaging Retiga R6 camera was used for all imaging. Olympus objectives were chosen and adjusted using each lens's cover slip correction collar to match the thickness of the glass substrates, thereby minimizing aberrations. To avoid unintentional polymerization, a 552 nm long‐pass filter was placed between the halogen lamp and the sample. The polarizer and analyzer were maintained in a crossed orientation, and the cell was rotated so its rubbing direction formed a 45° angle with the polarizer's transmission axis, creating a bright‐field configuration that was preserved for each measurement.

### 4.3 1 Photon Polymerization

An optical illumination arrangement was constructed around a 405 nm LED, selected because its emission wavelength overlaps with the absorption band of IR819 [64]. The light was collimated using a plano‐convex lens (LA1951‐A, Thorlabs; *f* = 25.4 mm) and patterned with a negative USAF test target (R1DS1N1, Thorlabs), whose rectangular features provided defined regions for localized polymerization and director‐state locking. A 2.5× microscope objective (Melles Griot; NA = 0.07) together with an aperture (ID25, Thorlabs) was incorporated into the projection system. To visualize and align the projected pattern on the LC device, an imaging system comprising a 50× objective (LMPlanFI, Olympus; NA = 0.5) and a CCD camera (DCU223M, Thorlabs) was employed. The LC cells were mounted on a translation stage for controlled positioning relative to the projected pattern and LED focal spot. Voltage control was applied using a waveform generator (MP750510, Multicomp Pro) to pre‐align the director configuration before localized polymerization.

### 4.4 2 Photon Polymerization

The two‐photon direct laser writing (2PP‐DLW) system was powered by a diode‐pumped solid‐state CW laser (Spectra‐Physics Millennia V, 532 nm), which pumped a Ti:Sapphire laser (780 nm, 100 fs pulses, 80 MHz). The output beam passed through a power‐control module consisting of a motorized half‐wave plate, polarizing beamsplitter, and beam trap. Rotation of the half‐wave plate enabled fine power tuning by polarization control, with excess light diverted to the trap. A 10:90 non‐polarizing beamsplitter and photodiode (Thorlabs S120C) provided real‐time power monitoring, while a fast shutter (Thorlabs SH05, 20 ms response) near the Fourier plane enabled synchronized beam gating with the translation stage. Beam conditioning was achieved with an adjustable half‐wave plate and expansion optics, allowing polarization optimization to mitigate birefringence‐induced aberrations. The beam was then directed by mirrors and a short‐pass dichroic mirror to the back aperture of the objective lens. The objective, mounted on a piezo scanner (PI PIFOC, 100 µm range), allowed depth control during fabrication. Sample positioning was provided by three‐axis translation stages (Aerotech ANT95XY‐050 and ANT95V‐3; 1 nm resolution), with cells secured on a custom 3D‐printed mount. Illumination was provided by a halogen lamp with a 550 nm filter, and imaging was performed using a CCD camera with a 200 mm lens, polarizer, and analyzer.

## Conflicts of Interest

The authors declare no conflict of interest.

## Supporting information




**Supporting File**: smtd70559‐sup‐0001‐SuppMat.docx.

## Data Availability

The data that support the findings of this study are available from the corresponding author upon reasonable request.
