# Supplementary material for: From Millimeters to Microns: A Hybrid Strategy for Reconfigurable Liquid‐Crystal Patterning
Source: Small Methods. 2026 Feb 18;10(5):e01892. doi: 10.1002/smtd.202501892 (PMC12972253; doi:10.1002/smtd.202501892)
Supplement: Supplementary file 1 — Supporting File: smtd70559‐sup‐0001‐SuppMat.docx. [file SMTD-10-e01892-s001.docx]

Supporting Information

From Millimeters to Microns: A Hybrid Strategy for Reconfigurable Liquid-Crystal Patterning

*Adithya Pradeep^1*^, Yunuen Montelongo^1^, Jun-Seok Ma^1,2^, Zhiyu Xu^1^, Tianxin Wang^1^, Ji Qin^1^, Camron Nourshargh^1^, Martin J. Booth, Steve J. Elston^1*^ and Stephen M. Morris^1 *^*

Adithya Pradeep, Yunuen Montelongo, Jun-Seok Ma, Zhiyu Xu, Tianxin Wang, Ji Qin, Camron Nourshargh, Martin J. Booth, Steve J. Elston, Stephen M. Morris

^1^Department of Engineering Science, University of Oxford, Parks Road, Oxford, OX1 3PJ, UK

E-mail: [adithya.nair@eng.ox.ac.uk](mailto:adithya.nair@eng.ox.ac.uk), [steve.elston@eng.ox.ac.uk](mailto:steve.elston@eng.ox.ac.uk), [stephen.morris@eng.ox.ac.uk](mailto:stephen.morris@eng.ox.ac.uk)

Jun-Seok Ma

^2^Nature Sciences Research Institute, KAIST, 291 Daehak-ro, Yuseong-gu, Daejeon 34141, Republic of Korea

**Aberration Correction and Optical Simulation for One-Photon Polymerization Patterning**The resolution achievable in the one-photon polymerization (1PP) system used for patterning in this work is strongly influenced by optical aberrations present in the imaging pathway ^[1-3]^. To demonstrate the significance of aberration correction in the patterning process, experimental observations and numerical simulations were conducted to assess how these distortions affect the projected pattern quality. Details of these approaches are provided in the following sections, and the impact of optical aberrations on feature clarity and edge sharpness is evaluated.

**Simulation Process**

A computational analysis was performed to examine distortions introduced during optical pattern projection in the one-photon polymerization (1PP) system developed for this work. These distortions, arising from aberrations along the optical path, can distort the projected pattern and reduce feature sharpness. To characterize and mitigate these effects, optical field propagation was simulated using the angular spectrum method ^[4–6]^, a Fourier-based technique for modeling scalar wavefront propagation ^[7]^. In this framework, the optical field at a given plane is Fourier transformed to the spatial-frequency domain, multiplied by the free-space propagation kernel for the specified distance, and then inverse transformed back to the spatial domain. The target pattern and the optical elements used in the experiment, such as lenses, were represented by complex transfer functions that modulate both amplitude and phase. Aberration terms were introduced through position-dependent phase maps at the pupil plane, and the resulting sample-plane intensity was compared with experiment.

The simulation process began with loading a grayscale image of the target pattern into MATLAB. Pixel values were converted to floating-point format and normalized to the [0, 1] range. The square root of these normalized intensities was taken to obtain the optical field’s amplitude distribution. To preserve consistent spatial orientation with the physical system, the resulting amplitude array was flipped vertically. Spatial sampling intervals along the *x* and *y*-axes were chosen to be ten times the laser wavelength (405 nm), which defined the physical scale of the simulation region. Coordinate arrays were generated such that the optical field was centered around the origin.

To capture source-induced phase fluctuations and the partial spatial coherence of the 405 nm LED, a randomized phase screen was generated and smoothed with a Gaussian low-pass filter ^[8]^. This phase mask was multiplied with the amplitude distribution to generate a complex input field containing both amplitude and phase information. The field was then propagated using the angular spectrum method in two steps: first from the object plane to an intermediate lens plane, and then from the lens plane to the final observation plane.

To incorporate lens effects, a spherical lens profile was introduced based on geometric parameters including radius of curvature and thickness. A corresponding two-dimensional (2D) phase map was generated to represent the spatially varying phase delay introduced by the lens material. This map was multiplied with the optical field at the lens plane, applying the focusing behavior of the lens to the wavefront. After propagation to the patterning plane, the resulting intensity distribution reflects the lens geometry, allowing the simulation to capture both free-space propagation and lens-induced focusing and distortions.

The simulations showed that slow phase variations in the projected light field, caused by imperfections in the lens or elsewhere in the optical path, blur the projected image. In particular, these variations smooth out the intensity profile and reduce the contrast at feature edges. This result highlights how optical aberrations can limit the resolution of the 1PP projection system and why explicit correction of such aberrations is necessary to achieve sharp, high-fidelity patterns.


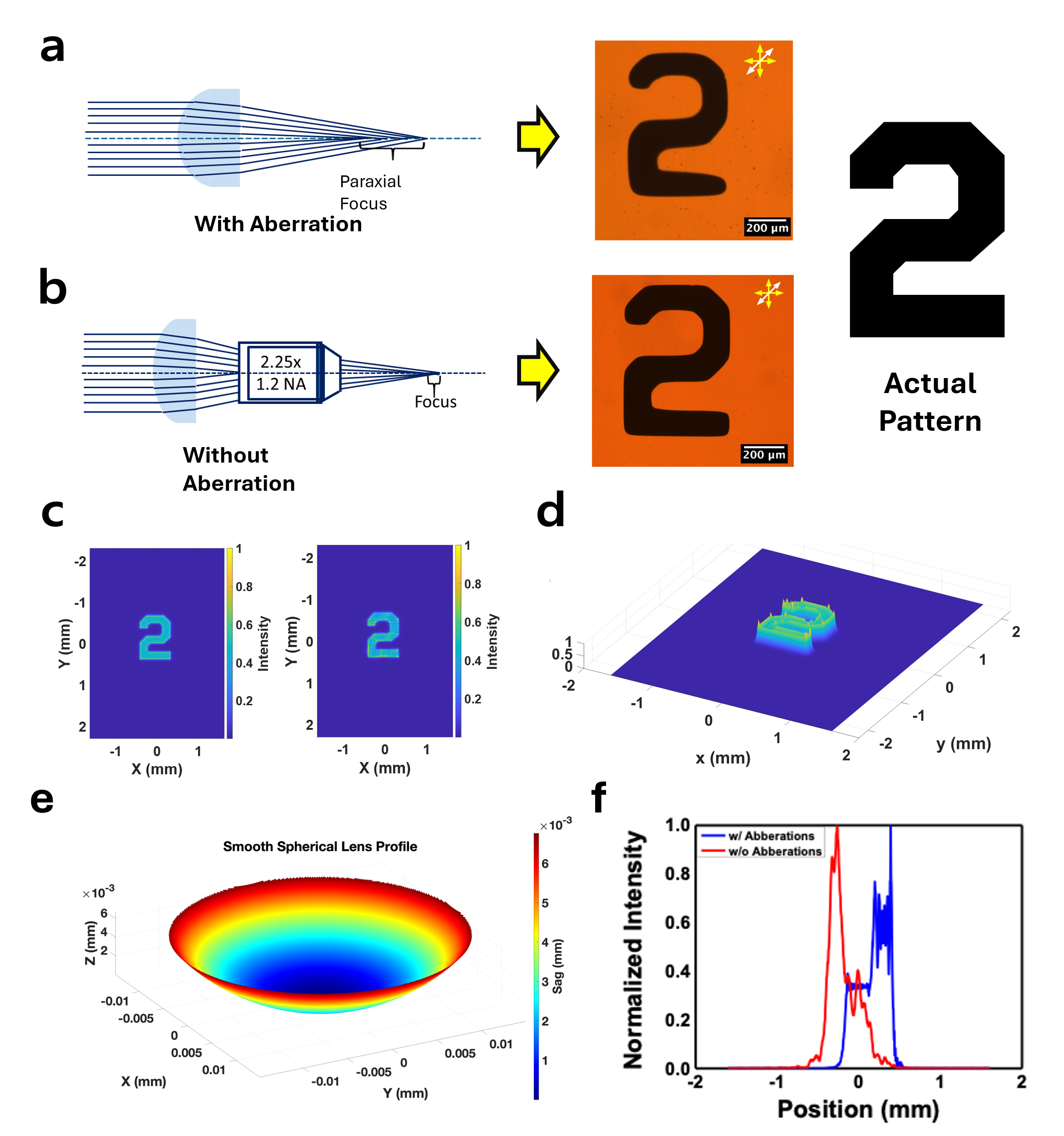


**Figure S1:** **(a)** Schematic representation of spherical aberration in a plano-convex lens. **(b)** Correction of spherical aberration using a high numerical aperture (0.07 NA) 2.5x microscope objective. **(c)** Experimental patterns of the number "2" observed in the LC cell under different conditions: with aberration, without aberration, and the ideal (target) pattern. **(d)** Simulated spherical lens surface profile. **(e)** Normalized intensity plots along the propagation axis, comparing aberrated (red) and non-aberrated (blue) conditions. **(f)** Simulated intensity distribution of the pattern: (left) 3D surface intensity plot of the aberrated "2" after propagation through a phase mask; (middle) normalized 2D field intensity for the aberrated case; and (right) normalized 2D field intensity for the non-aberrated case.

**Experimental Demonstration**

Experimentally, the relationship between the patterning process and spherical aberrations was assessed with comparative ray-propagation diagrams for two optical configurations, as shown in Figures S1(a) and (b). With a plano-convex lens alone the peripheral rays focus closer to the lens than paraxial rays, a characteristic of spherical aberration that leads to a blurred focal region. However, the microscope objective minimizes this effect, causing rays to converge to a much tighter, near-diffraction-limited focus. Figures S1(a) and S1(b) also illustrate the polymerized liquid crystal (LC) patterns obtained using different optical configurations. When only a plano-convex lens is used (Figure S1(a)), the projected “2” appears significantly distorted due to spherical aberration and non-uniform focus. The peripheral regions become especially skewed, creating a “shrunken” appearance at one end of the pattern. By contrast, introducing a 2.5× microscope objective with an NA of 0.07, along with an aperture, greatly reduces this aberration (Figure S1(b)). The polarizing optical microscopy (POM) images confirm that the “2” is far less distorted, with its edges more closely matching the intended design. These observations underscore that careful optical optimization, particularly lens selection, aperture placement, and alignment, is required to achieve high-fidelity LC patterning in the 1PP system.

**Phase Mask and Calculations**

Figure S1(e) shows the three-dimensional (3D) profile of the lens which was used as a phase mask in the simulation during the light propagation replicating the experiment with the plano convex lens. A simulation was performed that could visualize the 3D surface profile (sag) of a spherical lens with a given radius of curvature R and diameter D. The (*x*, *y*) domain was sampled on a uniform grid of N × N points spanning $\pm\frac{D}{2}$ .The standard sag equation ^[9]^,

$$Z=R-\sqrt{R^{2}-(x^{2}+y^{2})}$$

was used to compute the vertical displacement (i.e., sag) at each sampled point, while regions outside the lens aperture $x^{2}+y^{2}>{(D/2)}^{2}$ were masked out. The resulting 3D surface plot was rendered using a color map to illustrate variations in sag, providing a clear depiction of the lens’s curvature. This approach facilitated quick assessment of lens design parameters, allowing for verification of spherical symmetry and identification of any potential aberration-inducing features. Using the specified lens parameters, the lens surface was computed and confirmed to match the standard concave spherical profile. The associated phase map represents the lens delays at each point, clearly depicting a radially varying pattern. Areas of thicker lens material produce a higher phase shift, whereas thinner regions impart relatively lower shifts. After multiplying the field by the lens phase map and propagating again, the resulting intensity distribution at the observation plane demonstrated a focused spot, as expected from a simple spherical lens. The simulation produced a focused spot with a bright central maximum surrounded by rings of decreasing intensity, as expected for a simple spherical lens. These rings result from the focusing properties of the lens and are well captured by the angular spectrum method. In the 1PP process, this intensity profile determines the exposure dose: the central peak corresponds to a region of high light intensity, while the surrounding rings create weaker exposure halos. These halos broaden the polymer boundaries and reduce contrast at the feature edges in the LC cell.

Figure S1(c) shows 2D maps of the normalized field intensity for aberrated and non-aberrated conditions, provided for comparison with the POM images in Figures S1(a) and S1(b). The aberrated case (the right image) exhibits slight broadening and reduced contrast along the edges of the “2” pattern, indicative of the distortions introduced by optical aberrations. In contrast, the non-aberrated simulation (the left image) yields a sharper intensity profile with improved definition around the pattern boundaries. These comparisons underscore the importance of minimizing aberrations to enable accurate pattern projection and demonstrate the effectiveness of the lens design and correction strategies in creating well-defined intensity patterns after propagation. Figure S1(d) presents a 3D plot of the simulated intensity distribution, revealing a distinct “2” pattern emerging from the propagated optical light field. The intensity values are normalized to the maximum, emphasizing the regions of highest optical power within the formed image.

To assess the impact of phase (aberrations) on the projected light field profile, one-dimensional (1D) intensity profiles ^[10]^ were extracted from the centre row of the simulated 2D optical light field cross sections (Figure S1(c)). These profiles were then normalized relative to the maximum intensity. Full width at half maximum (FWHM) values were computed where the points were identified where intensity falls to 50% of its peak value and measures the distance between them.

Figure S1(f) compares simulated one-dimensional intensity profiles of the projected light field calculated with and without aberrations. In the aberrated case, the profile shows a broader full width at half maximum (FWHM) of 0.239 mm, while the corrected case gives a narrower FWHM of 0.166 mm, corresponding to an increase of about 44% in spot size when aberrations are present. These simulated FWHM values demonstrate that phase aberrations broaden the focal intensity distribution, whereas correcting them reduces the diameter at half-maximum and sharpens the focus.

Overall, this analysis was undertaken to optimize the one-photon projection step and to increase the attainable resolution of the 1PP mask-projection system. Simulations and POM observations show that uncorrected spherical aberration and low-frequency phase errors reduce the focusing of the projected light field, broaden the exposure profile, and distort the written features. Consequently, active aberration control is required to obtain tight focusing and accurate feature reproduction. The plano-convex lens alone produces distorted and compressed features because peripheral rays focus closer than paraxial rays. Using a 2.5× objective with NA 0.07 together with an aperture suppresses these errors, restores edge contrast, and enables rapid writing of large, accurate 1PP patterns. This corrected 1PP stage provides the high-quality outlines that are then combined with two-photon direct laser writing for high-resolution features in an LC device.


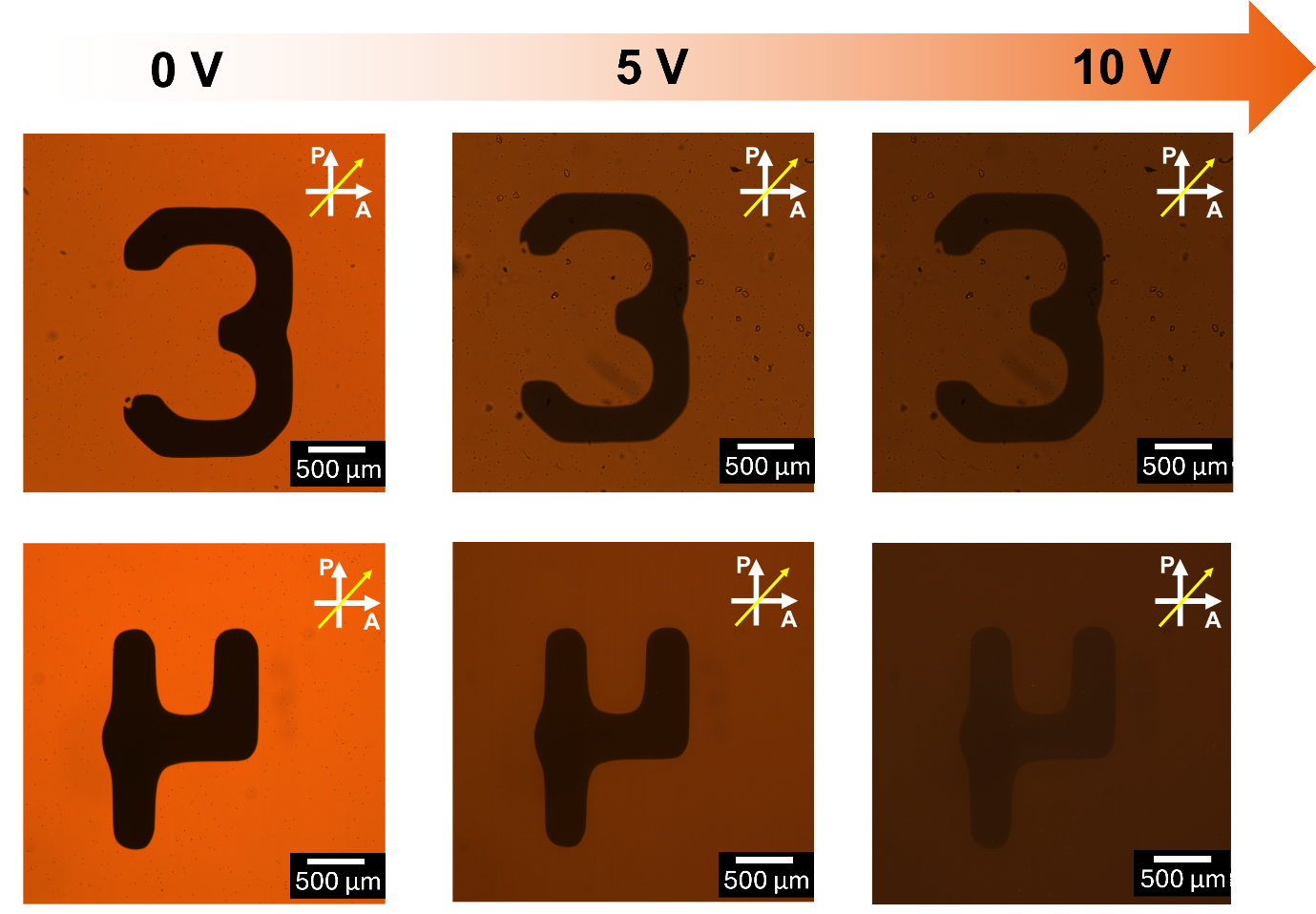


**Figure S2:** Formation and stabilization of bulk-projected patterns by 1PP under applied voltage. POM images show the same features at 0, 5, and 10 V (labels). The dark alphanumeric region corresponds to the 1PP-written area where the LC is locked in a homeotropic state; the brightness change with voltage occurs only in the unpolymerized background. During 1PP exposure, a voltage of 10 V was applied across the cell to set the homeotropic state in the written region. White arrows indicate the polarizer (P) and analyzer (A) orientations, and yellow arrows indicate the rubbing direction of the alignment layers. Scale bars 500 μm.


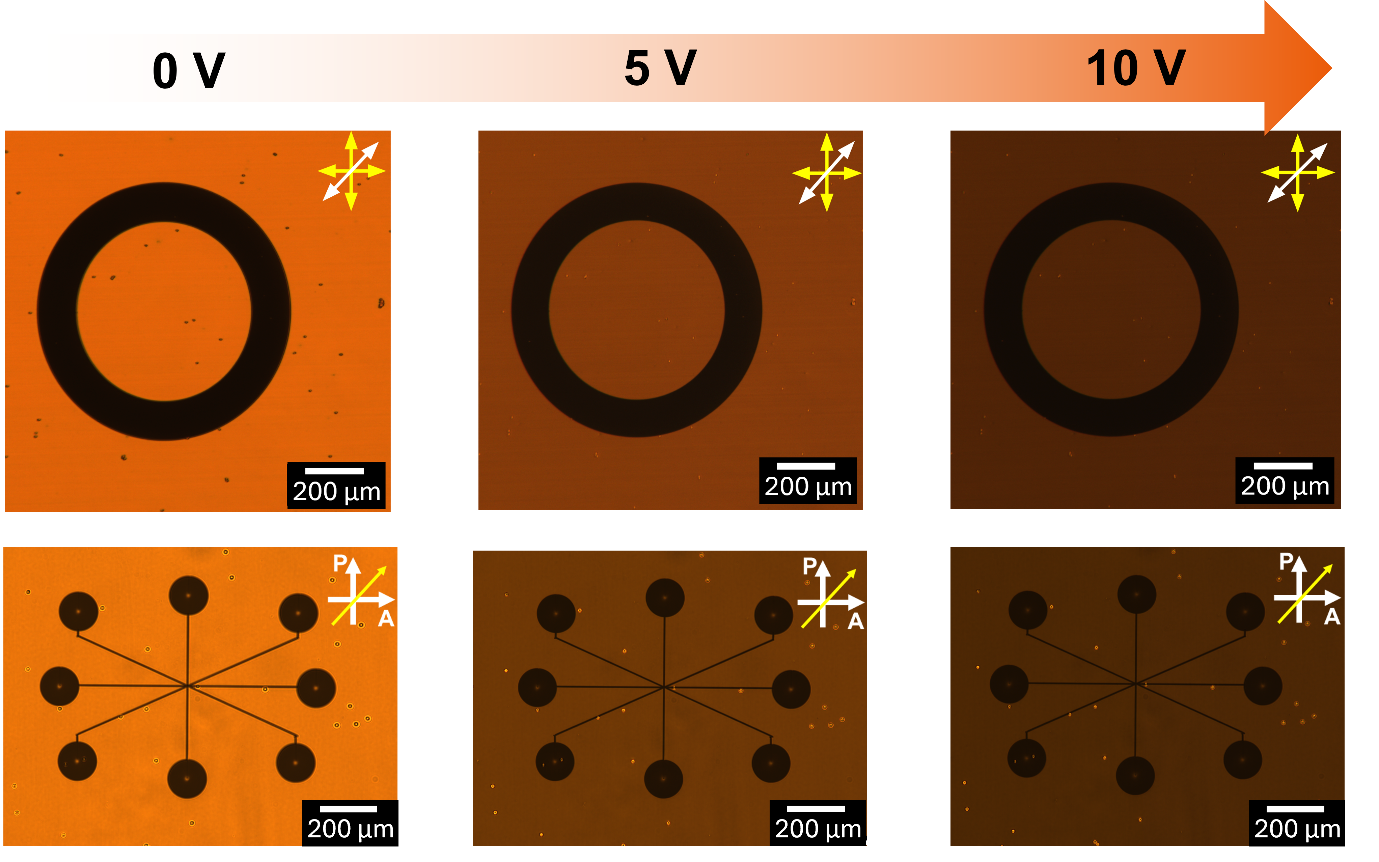


**Figure S3:** POM images showing localized polymer networks fabricated by two-photon polymerization (2PP) and their voltage response. During 2PP fabrication, voltage of 10V was applied across the cell to lock the director homeotropic within the written features. The same samples are then viewed at 0, 5, and 10 V (labels). The polymerized regions remain dark and shape-stable, while intensity changes with voltage occur only in the surrounding, non-polymerized LC. White arrows indicate the polarizer (P) and analyzer (A) orientations; yellow arrows indicate the rubbing direction. Scale bars are 200 μm.


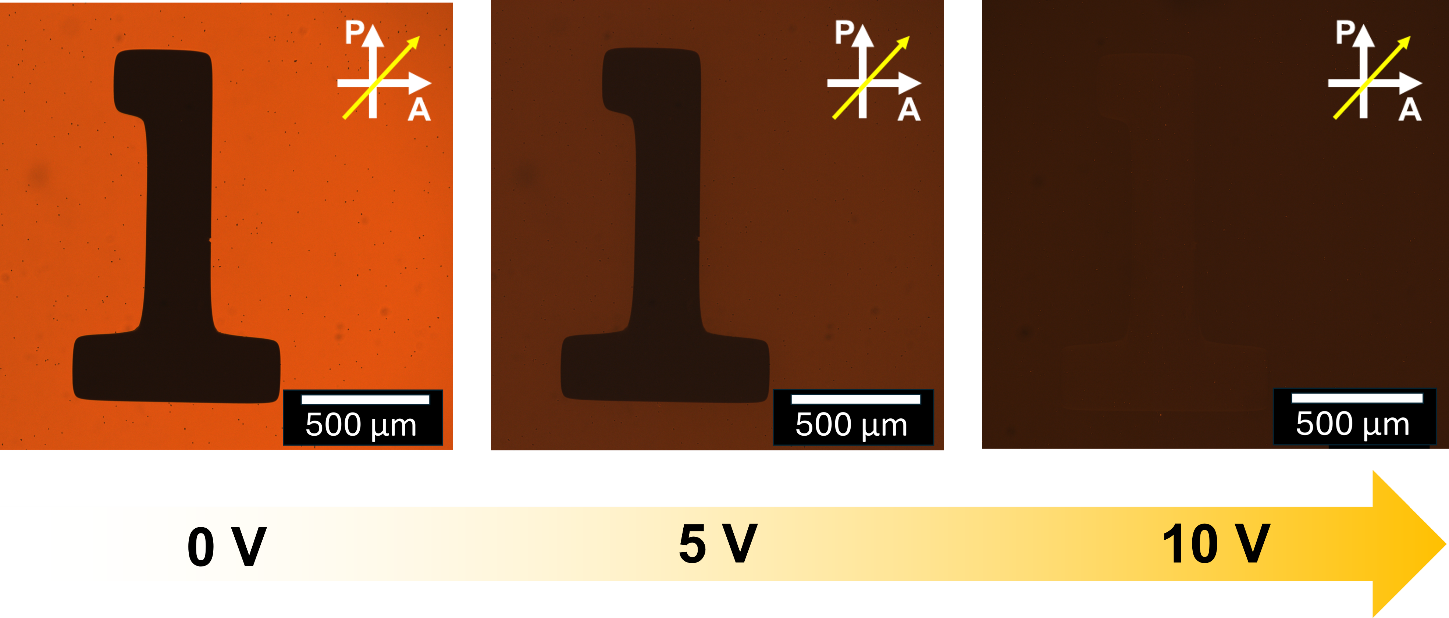
**Figure S4:** POM images showing voltage-activated optical cloaking in a cell patterned by 1PP.  During 1PP, voltage of 10V was applied across the cell to set a homeotropic state inside the written region. A sequence of images illustrating the modulation of pattern visibility with applied voltage. At low voltages, the patterned region (polymer stabilized homeotropic nematic region) remains visible due to refractive index mismatch between the patterned and surrounding (non-polymerized) regions. As the voltage increases, index matching occurs, leading to optical cloaking of the structure. Upon removal of voltage, the visibility of the pattern is restored. This demonstrates dynamic, reversible control over optical camouflage via voltage-tunable anisotropy. The polarizer (P) and analyzer (A) orientations are illustrated by the double-headed white arrows while the rubbing direction is illustrated by the double-headed yellow arrow. Scale bars are 500 μm.


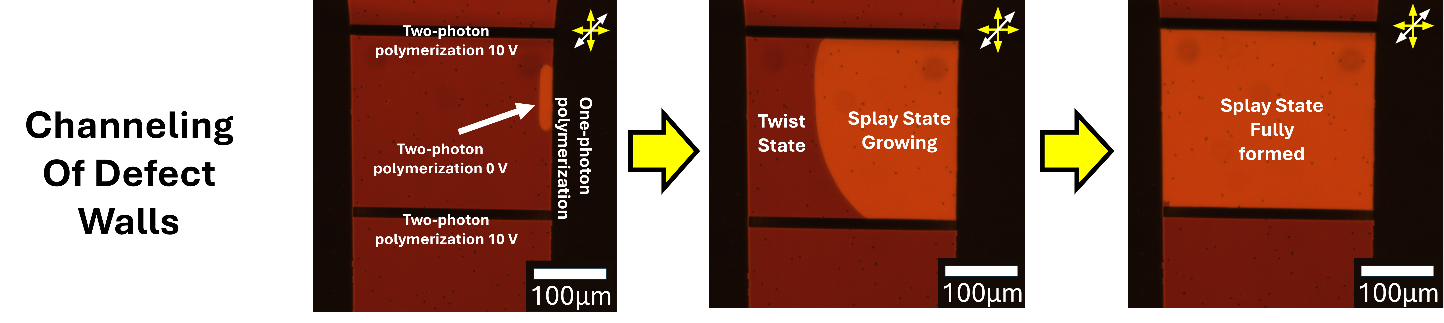


**Figure S5:** Directed splay defect transport using the hybrid 1PP–2PP patterning strategy. The left panel shows polymer walls formed by one-photon polymerization (1PP) on either side of the region, with a linear feature inscribed by two-photon polymerization (2PP) to define the channel in between. Upon laser inscription of this linear polymer network in the splay state, voltage modulation triggers anisotropic defect movement along the predefined channel. The middle panel shows the growth of the splay-state defect wall guided along the inscribed line, while the right panel shows the fully developed splay state confined within the channel. This demonstrates how the combined 1PP/2PP approach selective activation and enables programmable control over defect dynamics through spatially encoded anchoring and external field stimuli. The polarizer (P) and analyzer (A) orientations are illustrated by the double-headed white arrows while the rubbing direction is illustrated by the double-headed yellow arrow. Scale bars are 100 μm.


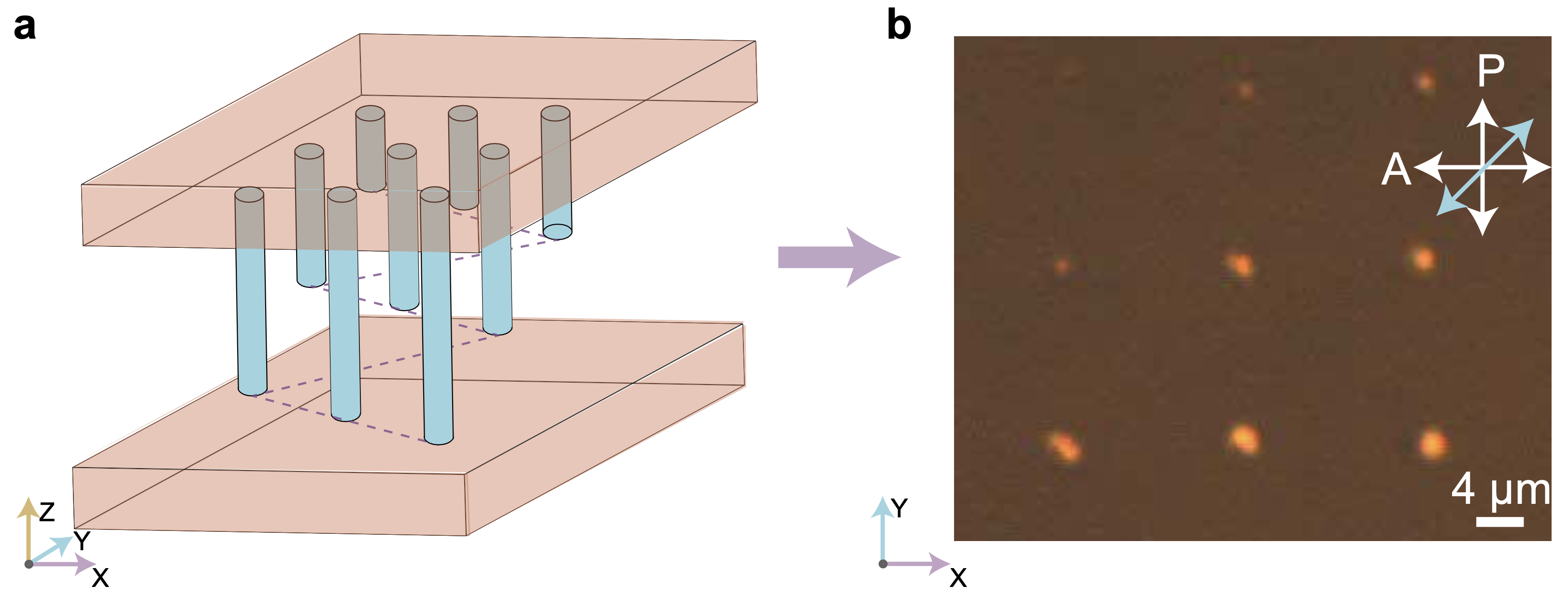


**Figure S6:** 3D pillar array illustrating depth control in 2PP patterning. (a) Schematic of a 2PP laser written polymer pillar array in a sandwich-cell geometry, where pillar heights are varied to demonstrate multi-level, out-of-plane micro-structuring from shallow to deeper features within the cell thickness. (b) Representative POM image of the corresponding fabricated array, showing optically distinct features at the pillar locations. The polarizer (P) and analyzer (A) orientations are illustrated by the double-headed white arrows while the rubbing direction is illustrated by the double-headed pink arrow. The scale bar is 4 µm.


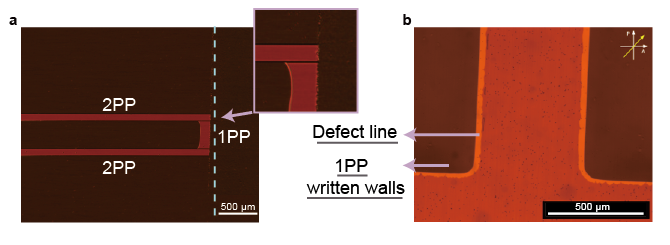


**Figure S7:** Independent 2PP writing adjacent to pre-polymerized 1PP regions and resulting defect guidance. (a) Polarizing optical microscopy (POM) image showing a 2PP-inscribed polymer line written in the vicinity of a pre-formed 1PP-polymerized region (dashed boundary), demonstrating local, maskless patterning without mechanically disturbing the previously polymerized structure (inset shows the junction detail). (b) POM image of a defect line forming and being guided within a nematic Pi-cell geometry by 1PP-written polymer walls that lock-in the surrounding director configuration. Scale bars are 500 µm.

**References**

[1] Wang, J. and Zhang, Y., 2021. Adaptive optics in super-resolution microscopy. *Biophysics Reports*, *7*(4), p.267.

[2] Booth, M., Andrade, D., Burke, D., Patton, B. and Zurauskas, M., 2015. Aberrations and adaptive optics in super-resolution microscopy. *Microscopy*, *64*(4), pp.251-261.

[3] Linfoot, E.H., 1957. Image quality and optical resolution. *Optica Acta: International Journal of Optics*, *4*(1), pp.12-16.

[4] He, Z., Sui, X., Jin, G. and Cao, L., 2019. Distortion-correction method based on angular spectrum algorithm for holographic display. *IEEE Transactions on Industrial Informatics*, *15*(11), pp.6162-6169.

[5] Atalar, A., 1978. An angular‐spectrum approach to contrast in reflection acoustic microscopy. *Journal of Applied Physics*, *49*(10), pp.5130-5139.

[6] Yang, J., Li, J., He, S. and Wang, L.V., 2019. Angular-spectrum modeling of focusing light inside scattering media by optical phase conjugation. *Optica*, *6*(3), pp.250-256.

[7] Goodman, J.W., 2005. *Introduction to Fourier optics*. Roberts and Company publishers.

[8] Stewart, W.C., Firester, A.H. and Fox, E.C., 1972. Random phase data masks: fabrication tolerances and advantages of four phase level masks. *Applied Optics*, *11*(3), pp.604-608.

[9] Lamontagne, F., Fuchs, U., Trabert, M. and Möhl, A., 2018. Aspheric lens mounting. *Optical Engineering*, *57*(10), pp.101708-101708.

[10] Xu, Y., Lin, H., Yi, F. and Liu, J., 2023. Design optimization of one-dimensional aberration-free x-ray gradually focusing lenses. *AIP Advances*, *13*(1).
